# Estimating HIV Incidence during Pregnancy and Knowledge of Prevention of Mother-to-Child Transmission with an Ad Hoc Analysis of Potential Cofactors

**DOI:** 10.1155/2016/7397695

**Published:** 2016-03-31

**Authors:** Thomas Obinchemti Egbe, Rose-Mary Asong Tazinya, Gregory Edie Halle-Ekane, Eta-Nkongho Egbe, Eric Akum Achidi

**Affiliations:** ^1^Department of Obstetrics and Gynecology, Douala General Hospital, Douala, Cameroon; ^2^Faculty of Health Sciences, University of Buea, Buea, Cameroon; ^3^Mbingo Baptist Hospital Annexe, Douala, Cameroon; ^4^District Hospital Poli, Poli, Cameroon; ^5^Faculty of Science, University of Buea, Buea, Cameroon

## Abstract

*Background*. We determined the incidence of HIV seroconversion during the second and third trimesters of pregnancy and ad hoc potential cofactors associated with HIV seroconversion after having an HIV-negative result antenatally. We also studied knowledge of PMTCT among pregnant women in seven health facilities in Fako Division, South West Region, Cameroon.* Method*. During the period between September 12 and December 4, 2011, we recruited a cohort of 477 HIV-negative pregnant women by cluster sampling. Data collection was with a pretested interviewer-administered questionnaire. Sociodemographic information, knowledge of PMTCT, and methods of HIV prevention were obtained from the study population and we did Voluntary Counselling and Testing (VCT) for HIV.* Results*. The incidence rate of HIV seroconversion during pregnancy was 6.8/100 woman-years. Ninety percent of the participants did not use condoms throughout pregnancy but had a good knowledge of PMTCT of HIV. Only 31.9% of participants knew their HIV status before the booking visit and 33% did not know the HIV status of their partners.* Conclusion*. The incidence rate of HIV seroconversion in the Fako Division, Cameroon, was 6.8/100 woman-years. No risk factors associated with HIV seroconversion were identified among the study participants because of lack of power to do so.

## 1. Background

Vertical transmission of Human Immunodeficiency Virus (HIV) is still a major challenge in the world, especially in the developing countries (USAID) [1]. It is estimated that 90% of HIV infections in children result from mother-to-child-transmission (WHO (Switzerland)) [2]. In the absence of any intervention to prevent MTCT (PMTCT), the MTCT rate varies between 13% and 48% [3, 4]. Maternal combination antiretroviral therapy (ART) together with postnatal interventions has demonstrated its efficacy in substantially reducing the risk of MTCT in African breastfed children to less than 5% (USAID 2013) [3, 5].

However, access to ART and the uptake of PMTCT programs remain limited and children continue to be HIV infected (Abidjan, Cote d'Ivoire) [2].

The World Health Organisation (WHO) guidelines recommend that all pregnant women should be tested for HIV in the first trimester and that a second test be considered in the third trimester by 34 weeks of gestational age [1, 6–8]. Guidelines in resource-limited settings are increasingly recommending HIV testing as early as possible during pregnancy and repeat testing towards the end of pregnancy or during labour, a strategy that has proven to be cost effective [8]. Despite these recommendations, recent studies show relatively high rates of seroconversion during pregnancy in Africa. Brubaker et al. report HIV incidence rates of 10.8% in serodiscordant couples in Kenya whenever a pregnancy occurred [9]. Keating et al. further report a lower seroconversion rate of 1% amongst pregnant women in Malawi [10]. More recently, a meta-analysis published in 2014 reports an aggregate seroconversion rate of 3.8 per 100 person-years in African countries by Drake et al. in Washington USA [11].

HIV testing during labour has remained a challenge over the years in Cameroon. In 2009, of the 94,406 women with a previous negative result who presented in the labour rooms of the clinics carrying out PMTCT activities, only 2,643 were retested, giving a proportion of 2.8% [12].

In Cameroon, the prevalence of HIV was estimated to be 4.3% in the general population; a serosurveillance survey among pregnant women showed an HIV prevalence of 7.6% in 2010 [13]. As a result, the number of new pediatric infections continues to grow in Cameroon and there are still thousands of new infections each year [14]. In 2011, the UNAIDS launched the Global Plan towards eliminating new HIV infections among children and keeping their mothers alive, making Cameroon, where overall MTCT risk was reported to be around 24% [1], one of the 21 priority countries.

Since 2011, Cameroon has tripled its coverage of PMTCT prophylaxis, ranging from 6.9% to 36.5% in 2011, leading to 30% fewer new HIV infections among children [15]. In 2011, Cameroon opted for the WHO Option A regimen for PMTCT prophylaxis. Continuing access of pregnant women living with HIV to prenatal HIV services and increasing access to HIV treatment for eligible children and pregnant women will reduce maternal and child mortality [15]. Cameroon has focused on strengthening PMTCT services and caring of pediatric HIV cases for the 2011–2015 period; 99.4% of health districts were equipped to provide HIV treatment services for pregnant women and children living with HIV in 2011. However, even where the most effective PMTCT interventions are available, many women and infants are lost at different steps of the PMTCT cascade [16]; and the low cumulative uptake of PMTCT services does not allow controlling the extent of MTCT in Cameroon. The HIV seroprevalence in the SWR is 11.9%. This is one of the highest in Cameroon, closely followed by the East Region with 9.3% [17].

There is a high rate of MTCT of HIV with seroconversion in pregnancy [10, 18]. In Cameroon, particularly in the South West Region, there are no reports regarding the incidence of HIV seroconversion during pregnancy. There is a probability that many cases that seroconvert in pregnancy go without appropriate management, resulting in high MTCT as reported by Muffih in 2011 [12].

Data on the seroconversion rate after initial negative HIV test result in pregnancy would be useful in improving the management of HIV in pregnancy in Fako Division, SWR, Cameroon.

The aim of this study was to determine the incidence of HIV seroconversion during the second and third trimesters of pregnancy and ad hoc potential cofactors associated with HIV seroconversion after having an HIV-negative test result in the booking visit. We also studied knowledge of PMTCT among pregnant women in seven health facilities in Fako Division, South West Region (SWR), Cameroon.

## 2. Materials and Methods

### 2.1. Study Design, Population, and Setting

This was a hospital based cohort study of women attending antenatal care (ANC) clinics and labour rooms of the maternity units of seven healthcare facilities in the Fako Division, South West Region, Cameroon, during the period between September 12 and December 4, 2011. Study participants were women who attended their booking or first antenatal care visit in any of the seven selected health facilities in the last six months and for whom an HIV test was done using the Determine test strips on this booking (first) ANC visit between 16 and 20 weeks of gestation. HIV seroconversion in pregnancy (HSP) was defined as maternal self-report of an HIV-negative test during the first antenatal care visit during this pregnancy, no documented use of antiretroviral drugs, and a positive HIV rapid test (Determine) done ≥3 months after the first antenatal care (booking) test.

The gestational age of the pregnancy was calculated from the last normal menstrual period (LNMP). All the women who were found to be HIV-negative at this first visit consented (written consent after study procedure and objectives had been explained to them) to participate in the study (Table 1). All study participants were counselled to repeat the HIV test within an interval of 3 to 6 months following the test of the booking ANC visit. All participants were those residing within a 10 Km radius from health facility for easy follow-up. The main outcome of interest was HIV seroconversion at second test during the ongoing pregnancy.

### 2.2. Sample Size and Sampling Procedure

Sample size was calculated by using the WHO-steps approach [19] with the assumptions of 95% confidence limits, 5% proportion of seroconverting women [18], and 2% margin of error. The minimum sample size was calculated to be 457 participants, but considering a nonresponse rate of 4%, we enrolled 477 participants for study.

A total of seven health facilities were selected by simple random sampling (balloting) for study. Participants who met the inclusion criteria were then selected by cluster sampling; and in each cluster, participants were included individually and consecutively to maximize confidentiality.

### 2.3. Data Collection Procedures

Data collection was done during a period of 12 weeks, from September 12 to December 4, 2011. During this period, the study participants were met at their various antenatal clinic sites on specific days of the week when these activities were carried out. A total of 4 weeks were spent per health facility with at least two facilities targeted at once depending on their ANC days (Table 2).

Information from each participant was collected through a pretested interviewer-administered survey questionnaire. Sociodemographic information (maternal age, gravidity, marital status and marital type, employment status, level of education, and residence), knowledge of PMTCT, and methods, if any, of HIV prevention were obtained from the study population.

Furthermore, Voluntary Counselling and Testing (VCT) for HIV was done on each study participant, whereby pretest counselling was done followed by the collections of 2-3 mL of venous blood by venipuncture into a dry vacutainer tube which was then allowed to settle for about 20 minutes to obtain serum from whole blood. The supernatant serum from the dry tube was then tested using Determine HIV-1/2 rapid test (Abbott Laboratories, Abbott Park, Illinois, USA) in the laboratory of the health facilities by trained laboratory personnel, experienced in HIV testing, according to manufacturer's test procedure. The same brand of test kits was used across all the health facilities that participated in the study. Following testing, a posttest counselling was done and results were delivered about one hour after testing. For participants who were positive for Determine HIV 1/2 rapid test, a second-line test, SD Bioline HIV 1/2 3.0 (Standard Diagnostics, Inc.), was done according to manufacturer's instructions to differentiate between HIV-1 and HIV-2.

A participant was only considered positive if both tests were positive, negative if Determine was negative, and indeterminate if Determine was positive and then Bioline test was negative [20–22].

All the participants who were diagnosed HIV-positive were treated according to Cameroon's national guidelines for the PMTCT [23], which at the time recommended Option B involving either AZT + 3TC + NVP or D4T + 3TC + NVP.

### 2.4. Data Management and Analysis

The Epi info 3.4.5 and Microsoft Excel 2010 software were used for statistical analysis. Numerical variables like age, parity, and gestational age were classified into groups and their frequencies expressed in percentage were presented; meanwhile, categorical variables like marital status, educational level, and occupation were expressed as frequencies. Comparison of seroconversion incidence to other variables was done using Fisher's exact test reported with *P* values that were considered significant if *P* was less than 0.05. Univariable analysis was done using logistic regression to identify the potential factors associated with seroconversion in pregnancy, and then those with a *P* value less than 0.2 were included in the final model for multivariable logistic regression. Results were reported as adjusted odds ratios (OR) together with their 95% confidence intervals (CI).

### 2.5. Ethical Consideration

Ethical clearance was obtained firstly from the Faculty of Health Sciences Institutional Review Board (FHS/IRB) as well as the Cameroon Baptist Convention (CBC) Institutional Review Board before patient enrolment. Then, an authorisation was obtained from the Regional Delegation of Public Health for the South West Region. Permission was obtained from the health districts and the various health facilities for the study to be carried out in the desired health facilities. A signed informed consent was also obtained from all the study participants. The respondents were only identified by registration numbers instead of names. All information obtained from respondents remained strictly confidential.

## 3. Results

### 3.1. Study Participants

#### 3.1.1. Characteristics of Study Population

*Table 1*. A total of 1954 antenatal women in seven healthcare facilities were tested for HIV to provide annual prevalence data in their first antenatal (booking) visit. Among this sample, 201 (10.3%) women were tested HIV-positive and were excluded from study. Amongst the remaining 1753 HIV-seronegative pregnant women, 477 (27.2%) were enrolled into the study in the second and third trimesters to study the incidence of seroconversion.

*Table 2*. The majority, 308 participants (64.65%), were in the age group 21–30 and 364 (76.32%) were married. About 350 (96.2%) of those who were married were monogamously married. Of the study population, 418 (87.6%) were urban dwellers, 394 (83.12%) had less than two children, three hundred and ninety (81.8%) were in the third trimester of pregnancy, and 388 (81.3%) had done secondary level of education and above.

**Figure 1:**
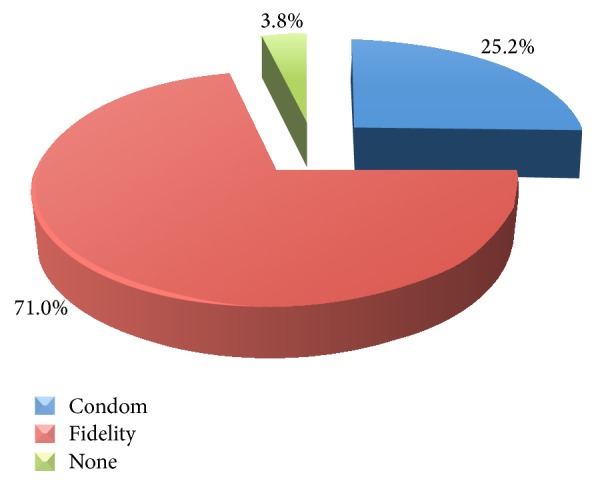
HIV prevention measures practiced by the participants.

*Figure 1*. Some women, 3.8%, had high risk behaviours. Most of the participants (99.6%) had one sexual partner.

### 3.2. Seroconversion Rates

*Table 3*. The incidence of participants who seroconverted was 2.1% (147.25 woman-years), giving an incidence rate of HIV seroconversion during pregnancy of 6.8 per 100 woman-years. The majority, 3% (8/262), of the patients who seroconverted did the second test 3 months after the first test.

*Table 4*. A minority, 31.9%, of participants did not know their HIV status prior to the first antenatal care (booking) visit while 33.0% of participants did not also know the HIV status of their partners/husbands.

*Table 5*. It shows that no statistically significant relationship was found between sociodemographic factors and HIV seroconversion in pregnancy.

**Figure 2:**
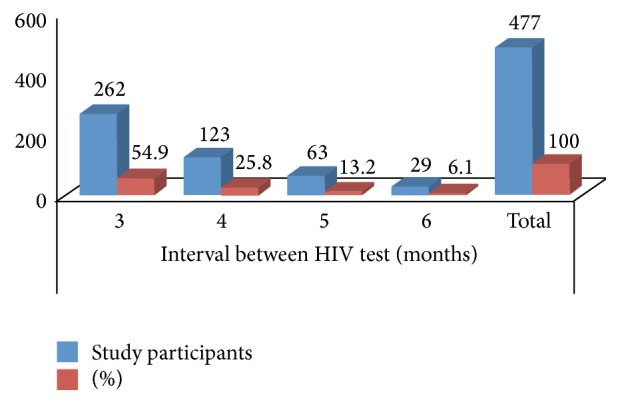
Time interval between repeat HIV test from booking test.

*Figure 2*. The majority (54.9%) of participants had a repeat test 3 months after the first negative (booking) test result while 25.8%, 13.2%, and 6.1% had their repeat test done at 4, 5, and 6 months, respectively.

Eighty percent (*n* = 8) of those who seroconverted did so by the fourth month after the booking or first antenatal care visit HIV testing (Figure 2). Six (60%) of those who seroconverted were in a monogamous regime and 3 (30%) were single, while 1 (10%) was in a polygamous regime (one husband and two wives).

*Table 6*. Among the 10 participants who seroconverted in pregnancy, 8 knew about MTCT and PMTCT of HIV including when transmission could occur (either during pregnancy, labour/delivery, and breastfeeding or when the mother does not know she is HIV-positive). All the eight participants also knew it was possible to prevent MTCT (*P* = 0.07) and 7 out of 8 knew at least one correct method of PMTCT (avoiding breastfeeding, taking antiretroviral treatment, caesarean delivery, or mother being aware of her HIV serologic status before engaging in a pregnancy) (*P* = 0.78). This was not statistically significant.

*Table 7*. All the participants who seroconverted were having sexual intercourse during the current pregnancy (*P* = 0.37) and 9 (90%) were not users of the condom. All the participants who were HIV-positive had only one sexual partner throughout pregnancy (*P* = 0.34). This was not statistically significant, *P* > 0.05.

*Table 8*. In multivariate analysis, the odds of HIV seroconversion during pregnancy were 5 times higher among pregnant women who did not know about PMTCT (aOR 5.4; 95% CI 1.06–27.56). Also, pregnant women who were employed were at a higher risk of seroconversion than those who were unemployed (aOR 3.2; 95% CI 0.68–15.4), though this was not statistically significant.

**Figure 3:**
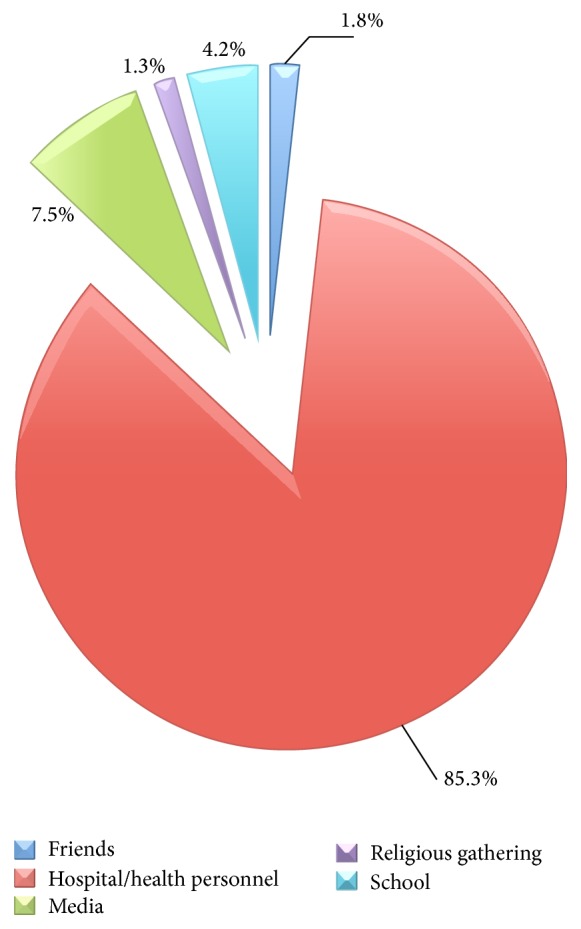
Source of information of participants regarding PMTCT.

*Figure 3*. The majority, 407 (85.3%), of the participants who knew about PMTCT got the information from the hospital/health personnel mainly during antenatal consultations, while 36 (7.5%) got informed through the media.

## 4. Discussion

HIV incidence during pregnancy and postpartum significantly increases risk of MTCT and is an important public health problem in Africa. Understanding maternal HIV incidence during this time period can be helpful to guide prevention and repeat testing strategies and policies, and little data on HIV incidence in pregnancy from West Africa exist. This study measures HIV incidence during pregnancy in seven healthcare facilities in Fako Division, South Region of Cameroon, by repeat testing later in pregnancy.

Women in the South West Region, like those in most other low-income countries, come to health facilities for antenatal care very late in pregnancy, usually in the second trimester [24]. At the same time, they prefer to give birth in a health facility because they perceive labour and delivery as a time of significant health risks that require biomedical attention [24]. For this reason we sometimes find it difficult to have women tested for HIV in the first trimester of pregnancy, especially in the rural areas.

### 4.1. Incidence of HIV Seroconversion in Pregnancy

The incidence of HIV seroconversion during pregnancy in this study was 2.1% (147.25 woman-years), yielding an incidence rate of 6.8/100 woman-years. These results are lower than that reported by Moodley et al. in 2009 in Uganda who showed that, among 2377 HIV-negative women retested, 1099 (46.2%) and 1278 (53.4%) were tested at urban and rural health facilities, respectively. Seventy-two women (3%) were HIV-positive (679 woman-years of exposure), yielding an HIV incidence rate of 10.7/100 woman-years (95% confidence interval (CI) 8.2–13.1) [18]. HIV incidence in pregnancy was higher but not statistically significant at the urban facilities (12.4/100 woman-years versus 9.1/100 woman-years) and at least two-fold higher among the 25–29- and 30–34-year age groups (3.8 and 4.5%, resp.) as compared with the less than 20-year age group (1.9%). Single women were at 2.5 times higher risk of seroconverting during pregnancy (*P* = 0.017) [18]. In another study, Humphrey et al. reported that breastfeeding associated transmission for mothers who seroconverted postnatally (*n* = 334) averaged 34.56 infant infections per 100 child-years (95% CI 26.60 to 44.91) during the first nine months after maternal infection, declined to 9.50 (95% CI 3.07 to 29.47) during the next three months, and was zero thereafter. Among women who seroconverted postnatally and in whom the precise timing of infection was known (≤90 days between last negative and first positive test) (*n* = 51), 62% (8/13) of transmissions occurred in the first three months after maternal infection and breastfeeding associated transmission was 4.6 times higher than in mothers who tested HIV-positive at baseline and whose infant tested HIV-negative with PCR at six weeks [25]. A 5% seroconversion incidence was reported in Zimbabwe in 2002 (unpublished data).

Our results conform with the 2.2% seroconversion rate reported by Qolohle et al. in 1995 at the King Edward VIII hospital in Durban, South Africa (2.6%) [26] and the 2.9% reported by Lockman and Creek in Francistown, Botswana, at 62 weeks postpartum [27].

With the 2.1% incidence of HIV or 6.8/100 woman-years incidence rate of HIV seroconversion during pregnancy in Fako Division, there would be a correspondingly increasing rate of MTCT of HIV, thereby increasing the pediatric HIV burden [28]. It has been estimated that 19% of infants born to HIV-positive mothers are HIV infected when tested at 19 months [29]. The percentage is even higher in cases of seroconversions in pregnancy. This high rate of infant infection can be attributed to the fact that maternal HIV infection during pregnancy and breastfeeding occasion equally high rates of MTCT of HIV-1.

All participants who seroconverted did so in the third trimester of pregnancy, when most of the study participants (81.8%) were recruited.

All the participants who seroconverted in our study had HIV-1 which is the type of HIV mainly responsible for MTCT of the virus [30]. Therefore, all cases of HIV seroconversion during pregnancy should be considered as potential risks for more pediatric HIV. Seventy percent of the participants who seroconverted were married; therefore, marriage does not protect women from HIV infection in pregnancy. This finding is in conformity with results obtained in Harare, Zimbabwe [31].

Eighty percent of the participants who seroconverted had 3-4-month interval between the two tests, while there was no case of seroconversion among women who had 6-month interval. These results differ from those obtained in the Tygerberg Hospital of Cape Town where it was reported that women especially at risk of seroconversion are those who book much earlier, thereby increasing the interval between the initial test at first ANC visit and the repeat test [29]. To identify cases of seroconversion more early, it will be advisable for repeat HIV testing to be done to pregnant women every three months until delivery, no matter the time of first ANC visit.

### 4.2. Risk Factors of HIV Seroconversion in Fako Division

The risk factors that were studied (age, marital status, residence, parity and knowledge of MTCT of HIV, HIV prevention, and HIV seroconversion in pregnancy) did not show any significant statistical variations. These results are not in conformity with those reported in Harare, Zimbabwe, where women aged 17 and below were at higher risk of seroconverting than the general population of HIV-negative pregnant women studied [31].

### 4.3. Knowledge of PMTCT in the Fako Division

In this study, 442 (98.2%) of participants who remained seronegative and 8 (1.8%) of those who seroconverted had a good knowledge of PMTCT techniques. This is probably because it has been a routine practice at all antenatal clinics to give the women talks on HIV and PMTCT, especially during the first ANC visit. Familiarity level with PMTCT practices in this study is similar to that obtained in Yaounde, Cameroon, in 2011 [32].

The majority of study participants, though aware of MTCT of HIV during pregnancy, did not take the necessary preventive measures. This was exemplified by the fact that some participants did not know their HIV status before the first antenatal care (booking) visit. This may be the result of inadequate sensitization of the population of the SWR or negligence on the part of the participants. This fact alone could have a negative impact on the PMTCT strategies put in place in Fako Division and based on recommendations of the WHO and the Government of Cameroon [7].

The limitations to this study include the fact that we did not study the male partners' or husbands' risk factors and relied on results of HIV tests done at the booking or first antenatal care visit in the different health facilities. Such tests could have produced faulty results. Also, seroconversions were determined using rapid tests, not HIV RNA tests, thus potentially underestimating incidence rates. Furthermore, the study was underpowered to detect cofactors for risk and only measured cofactors based on demographics and PMTCT knowledge. We did not assess many other potential cofactors for acquisition of HIV, such as STIs. Finally, the collection of data using an interviewer-administered questionnaire could have resulted in social desirability bias.

## 5. Conclusion

The incidence of HIV seroconversion among pregnant women in the study is 2.1%, yielding an incidence rate of 6.8/100 woman-years in Fako Division. Most of the participants seroconverted 3 months after the first test and therefore make up a potential risk for pediatric HIV.

The study was underpowered to study associated risk factors for seroconversion. Furthermore, there was adequate knowledge of PMTCT among pregnant women. Testing partners of pregnant women could be a major PMTCT strategy in our setting. Lastly, pregnancy did not stop the women from sexual activities and most of them had one sex partner.

## Figures and Tables

**Table 1:** Prevalence of HIV among pregnant women at first (booking) antenatal care visit in the various health facilities used for the study.

Health facility	Number of women tested	Number positive	Number negative	Percentage of positive cases
Baptist Hospital Mutengene	559	83	476	14.8
CDC Tiko Cottage Hospital	97	13	84	13.4
CDC Camp 7 Clinic Tiko	83	13	70	15.6
Down Beach Health Centre Limbe	504	27	477	5.4
Buea Road Health Centre	269	26	243	9.7
Buea Medicalised Health Centre	165	10	155	6.1
Limbe Regional Hospital	277	29	248	10.5

Total	**1954**	**201**	**1753**	**10.3**

**Table 2:** Demographic characteristics of the study population (*n* = 477).

Variable	Category	Frequency	Percentage (%)
Age group (years)	<26	235	49.3
	26–35	215	45.1
	>35	27	5.7
	Total	**477**	**100**

Marital status	Married	364	76.3
	Single	113	23.7
	Total	**477**	**100**

Marriage type	Monogamy	350	96.2
	Polygamy	14	3.8
	Total	**364**	**100**

Parity	0–2	394	83.1
	3-4	62	13.1
	>4	18	3.8
	Total	**474**	**100.0**

Gestational age	Second trimester (13–28 weeks)	87	18.2%
	Third trimester (29–40 weeks)	390	81.8%
	Total	**477**	**100%**

Level of education	Primary or less	144	30.3
	Secondary or more	332	69.7
	Total	**476**	**100.0**

Employment status	Employed	265	55.6
	Unemployed	212	44.4
	Total	**477**	**100.0**

**Table 3:** Incidence of HIV seroconversion in the seven healthcare facilities.

Number of participants	Time span between 1st and 2nd test (months)	Number of participants who seroconverted	Percentage of participants who seroconverted	Woman-years follow-up
262	3.0	8.0	3.1	65.5
123	4.0	1.0	0.81	41.0
63	5.0	1.0	1.59	26.25
29	6.0	0.0	0.00	14.5

Total = 477				147.25

Incidence of HIV seroconversion: 2.1% (147.25 woman-years).

Incidence rate of HIV seroconversion: 6.8/100 woman-years.

**Table 4:** Participants' knowledge of their HIV statuses and those of their partners before first antenatal care (booking) visit.

Variable	Category	Frequency	%
Did you know your HIV status before this pregnancy?	No	152	31.9
	Yes	324	68.1
	Total	**476**	**100**

Do you know your husband's/partner's HIV status?	No	157	33.0
	Yes	319	67.0
	Total	**476**	**100**

**Table 5:** Association between sociodemographic factors and HIV seroconversion in pregnancy.

Variable	Category	Negative *N* (%)	Positive *N* (%)	*P* values
Age group (in years)	<26	227 (97.8%)	5 (2.2%)	1.00
	26–35	208 (97.7%)	5 (2.4%)	
	>35	27 (100%)	0 (0.0%)	

Marital status	Unmarried	109 (97.3%)	3 (2.7%)	0.71
	Married	353 (98.1%)	7 (1.9%)	

Gestational age	13–28 (2nd trimester)	87 (100%)	0 (0.0%)	0.22
	29–40 (3rd trimester)	375 (97.4%)	10 (2.6%)	

Parity	0–2	381 (97.4%)	10 (2.6%)	0.58
	3-4	60 (100%)	0 (0.0%)	
	>4	18 (100%)	0 (0.0%)	

Level of education	Primary and below	138 (97.2%)	4 (2.8%)	0.50
	Secondary and above	323 (98.2%)	6 (1.8%)	

Employment status	Unemployed	208 (99.1%)	2 (1.0%)	0.2
	Employed	254 (97.0%)	8 (3.1%)	

Interval between HIV tests	≤4 months	372 (97.6%)	9 (2.4%)	0.70
	>4 months	90 (98.9%)	1 (1.1%)	

*P* values are based on Fisher's exact test.

**Table 6:** Association between knowledge of PMTCT and seroconversion.

Variable	Category	Negative *N* (%)	Positive *N* (%)	*P* values
Do you know about mother-to-child transmission of HIV?	No	20 (90.9)	2 (9.1)	0.07
	Yes	442 (98.2)	8 (1.8)	

If yes, where did you hear about it?	Friend	8 (100)	0 (0.0)	0.7
	Hospital/health personnel	377 (98.2)	7 (1.8)	
	Media	32 (97.0)	1 (3.0)	
	Religious gathering	6 (100)	0 (0.0)	
	School	19 (100)	0 (0.0)	

When does mother-to-child transmission take place?	Correct response	396 (98.0)	8 (2.0)	1.0
	Wrong response	46 (100)	0 (0.0)	

Is it possible to prevent mother-to-child transmission of HIV?	No	40 (100)	0 (0.0)	1.0
	Yes	402 (98.1)	8 (1.9)	

If yes, by what means?	Wrong response	28 (96.6)	1 (3.4)	0.78
	Correct response	374 (98.2)	7 (1.8)	

*P* values are based on Fisher's exact test.

PMTCT: prevention of mother-to-child transmission.

**Table 7:** Association between HIV prevention practices and HIV seroconversion during pregnancy in a group of 477 pregnant women in Fako Division, Cameroon.

Variable	Category	Negative *N* (%)	Positive *N* (%)	*P* values
Which HIV prevention measure do you practice?	None	17 (94.4)	1 (6.2)	0.42
	Condom	117 (98.3)	2 (1.7)	
	One sex partner	327 (97.9)	7 (2.1)	

Did you know your HIV status before this pregnancy?	No	146 (97.3)	4 (2.7)	0.73
	Yes	315 (98.1)	6 (1.9)	

Do you know your partner/husband's HIV status?	No	152 (97.4)	4 (2.6)	0.74
	Yes	309 (98.1)	6 (1.9)	

Have you been having sex during this pregnancy?	No	70 (100)	0 (0.0)	0.37
	Yes	391 (97.5)	10 (2.5)	

If yes, have you had sex with another man other than your husband?	No	390 (97.5)	10 (2.5)	1.0
	Yes	1 (100)	0 (0.0)	

Did you use the condom?	No	376 (97.6)	9 (2.4)	0.34
	Yes	15 (93.8)	1 (6.2)	

*P* values are based on Fisher's exact test.

HIV: human immunodeficiency virus.

**Table 8:** Risk factors for HIV seroconversion in a multivariate model.

Variable	aOR	95% CI	*P* value
Employment status			
Employed	3.2	0.68–15.4	0.142
Unemployed	1.0	—	
Do you know about MTCT of HIV?			
Yes	1.0	—	
No	5.4	1.06–27.56	0.042

Prev.: prevalence within a group (row); aOR: adjusted odds ratios; CI: confidence interval; MTCT: mother-to-child transmission.

## References

[1] Sidibé M. (2014). *Global Plan towards the Elimination of New HIV Infections among Children by 2015 and Keeping Their Mothers Alive*.

[2] De Cock K. M., Fowler M. G., Mercier E. (2000). Prevention of mother-to-child HIV transmission in resource-poor countries: translating research into policy and practice. *The Journal of the American Medical Association*.

[3] Leroy V., Ekouevi D. K., Becquet R. (2008). 18-Month effectiveness of short-course antiretroviral regimens combined with alternatives to breastfeeding to prevent HIV mother-to-child transmission. *PLoS ONE*.

[4] http://www.unaids.org/sites/default/files/en/media/unaids/contentassets/documents/epidemiology/2013/gr2013/UNAIDS_Global_Report_2013_en.pdf.

[5] Tonwe-Gold B., Ekouevi D. K., Viho I. (2007). Antiretroviral treatment and prevention of peripartum and postnatal HIV transmission in West Africa: evaluation of a two-tiered approach. *PLoS Medicine*.

[6] Rollins N., Mzolo S., Moodley T., Esterhuizen T., van Rooyen H. (2009). Universal HIV testing of infants at immunization clinics: an acceptable and feasible approach for early infant diagnosis in high HIV prevalence settings. *AIDS*.

[7] WHO PMTCT Guidelines 2014. https://www.google.cm/?gws_rd=cr&ei=Y5Q4VvvuIaf9ywOBzoK4DQ#q=who+pmtct+guidelines+2014.

[8] Tsague L., Abrams E. J. (2014). Commentary: antiretroviral treatment for pregnant and breastfeeding women—the shifting paradigm. *AIDS*.

[9] Brubaker S. G., Bukusi E. A., Odoyo J., Achando J., Okumu A., Cohen C. R. (2011). Pregnancy and HIV transmission among HIV-discordant couples in a clinical trial in Kisumu, Kenya. *HIV Medicine*.

[10] Keating M. A., Hamela G., Miller W. C., Moses A., Hoffman I. F., Hosseinipour M. C. (2012). High hiv incidence and sexual behavior change among pregnant women in lilongwe, malawi: implications for the risk of hiv acquisition. *PLoS ONE*.

[11] Drake A. L., Wagner A., Richardson B., John-Stewart G. (2014). Incident HIV during pregnancy and postpartum and risk of mother-to-child HIV transmission: a systematic review and meta-analysis. *PLoS Medicine*.

[12] Muffih T. P. (2009). *Aids Care and Prevention: Program Annual Report*.

[13] http://www.plateforme-elsa.org/wp-content/uploads/2014/05/Document_plaidoyer_MARPS_Affirmative-action.pdf.

[14] Penazzato M., Bendaud V., Nelson L., Stover J., Mahy M. (2014). Estimating future trends in paediatric HIV. *AIDS*.

[15] 2013 Progress Report on the Global Plan. http://www.unaids.org/sites/default/files/en/media/unaids/contentassets/documents/unaidspublication/2013/20130625_progress_global_plan_en.pdf.

[16] Tudor Car L., Brusamento S., Elmoniry H. (2013). The uptake of integrated perinatal prevention of mother-to-child HIV transmission programs in low- and middle-income countries: a systematic review. *PLoS ONE*.

[17] Cameroon National EMTCT Plan 2012. https://www.google.cm/search?sclient=psy-ab&site=&source=hp&q=Cameroon+national+EMTCT+plan+2012&oq=Cameroon+national+EMTCT+plan+2012&gs_l=hp.12...5474.5474.3.8379.1.1.0.0.0.0.0.0..0.0....0...1c.1.64.psy-ab..33.0.0.0.iSHl3-g6ofA&pbx=1&bav=on.2,or.r_cp.&bvm=bv.113370389,d.bGg&biw=1280&bih=358&dpr=1&ech=1&psi=oESzVsjfIIj6swGj3Y-oDw.1454590292157.11&ei=oESzVsjfIIj6swGj3Y-oDw&emsg=NCSR&noj=1.

[18] Moodley D., Esterhuizen T. M., Pather T., Chetty V., Ngaleka L. (2009). High HIV incidence during pregnancy: compelling reason for repeat HIV testing. *AIDS*.

[19] Eng J. (2003). Sample size estimation: how many individuals should be studied?. *Radiology*.

[20] http://apps.who.int/iris/bitstream/10665/179521/1/WHO_HIV_2015.15_eng.pdf?ua=1.

[21] Evans C., Ndirangu E. (2009). The nursing implications of routine provider-initiated HIV testing and counselling in sub-Saharan Africa: a critical review of new policy guidance from WHO/UNAIDS. *International Journal of Nursing Studies*.

[22] WHO Statement on HIV testing and counseling: WHO, UNAIDS re-affirm opposition to mandatory HIV testing. http://www.who.int/hiv/events/2012/world_aids_day/hiv_testing_counselling/en/.

[23] Guide de Poche. http://www.remed.org/GUIDE_DE_POCHE_PTME_recommandations_PNLS_Cameroun_09.pdf.

[24] Myer L., Harrison A. (2003). Why do women seek antenatal care late? Perspectives from rural South Africa. *Journal of Midwifery & Women's Health*.

[25] Humphrey J. H., Marinda E., Mutasa K. (2010). Mother to child transmission of HIV among Zimbabwean women who seroconverted postnatally: prospective cohort study. *BMJ*.

[26] Qolohle D. C., Hoosen A. A., Moodley J., Smith A. N., Mlisana K. P. (1995). Serological screening for sexually transmitted infections in pregnancy: is there any value in re-screening for HIV and syphilis at the time of delivery?. *Genitourinary Medicine*.

[27] Lockman S., Creek T. (2009). Acute maternal HIV infection during pregnancy and breast-feeding: substantial risk to infants. *Journal of Infectious Diseases*.

[28] Johnson L. F., Stinson K., Newell M.-L. (2012). The contribution of maternal HIV seroconversion during late pregnancy and breastfeeding to mother-to-child transmission of HIV. *Journal of Acquired Immune Deficiency Syndromes*.

[29] Theron G. B., Schoeman J., Carolus E. (2006). HIV seroconversion during pregnancy in the Tygerberg region of Cape Town. *South African Medical Journal*.

[30] Nkenfou C. N., Lobé E. E., Ouwe-Missi-Oukem-Boyer O. (2012). Implementation of HIV early infant diagnosis and HIV type 1 RNA viral load determination on dried blood spots in Cameroon: challenges and propositions. *AIDS Research and Human Retroviruses*.

[31] Mbizvo M. T., Kasule J., Mahomed K., Nathoo K. (2001). HIV-1 seroconversion incidence following pregnancy and delivery among women seronegative at recruitment in Harare, Zimbabwe. *Central African Journal of Medicine*.

[32] Zoung-Kanyi Bissek A.-C., Yakana I. E., Monebenimp F. (2011). Knowledge of pregnant women on mother-to-child transmission of HIV in Yaoundé. *Open AIDS Journal*.

